# The Merit-based Incentive Payment System: Pearson’s Chi-Square and Categorical Dependent Variable Models Analyzed for Domains—Effective Clinical Care and Efficiency/Cost Reduction

**DOI:** 10.36469/jheor.2021.29971

**Published:** 2021-12-06

**Authors:** Amrita Shenoy

**Affiliations:** 1 Healthcare Administration Program, School of Health and Human Services, College of Public Affairs University of Baltimore

**Keywords:** pay for performance program, quality measure types, national quality strategy (nqs) domains, merit-based incentive payment system, outcome measures

## Abstract

**Background:** Following the 2015 repeal of the Sustainable Growth Rate formula, the US Centers for Medicare & Medicaid Services’ formula under which physicians were reimbursed, two payment systems were put in place to incentivize physicians, one of which was the Merit-based Incentive Payment System (MIPS). MIPS emphasizes high-quality care that is accessible, affordable, and supports a healthier population.

**Objectives:** This research aims to measure characteristics of MIPS relevant to National Quality Strategy (NQS) domains, quality measure types, and clinical specialties; categorize MIPS with NQS domains and quality measure types by MIPS specialty types; and quantify the relationship between MIPS specialties, measure types, and two NQS domains, Effective Clinical Care (ECC) and Efficiency/Cost Reduction (E/CR), for years 2017 through 2020.

**Methodology:** The Pearson’s chi-square test examined distributions of the analyzed categorical variables. The Categorical Dependent Variable Method examined the association between the dependent and independent variables.

**Results:** The Pearson’s chi-square test showed statistically significant distributions between ECC and E/CR when analyzed with the types of quality measures. There were more process measures (93.81% vs 89.64% [*P*=.000]) in 2018 versus 2017. This changed minutely with significantly less process measures (93.75% vs 93.81% [*P*=.000]) in 2019 versus 2018. Finally, measure types changed minutely but significantly with less process measures (93.81% vs 93.75% [*P*=.000]) in 2020 versus 2019. The regression model showed that ECC was significantly associated with outcome measures through all analyzed years of this research.

**Conclusion:** The above findings show scope for including additional outcome measures, given its importance in MIPS. There is potential to increase the percentage allocation for reporting more outcome measures in quality. This re-allotment infers reporting more outcome measures aligning with priority outcome measures (PROMs). Re-allocating the incentive formula to report more outcome measures aligned with PROMs shows potential to increase reporting of more outcome measures under MIPS.

## INTRODUCTION

Providing high-quality care is one of the foremost goals of the health care system.^1^ In general, quality care that is accessible, affordable, and provides health benefits to the population has been one of the overarching goals of many US health care programs.^1^ These programs include the Merit-based Incentive Payment System (MIPS), the Hospital-Acquired Condition Reduction Program, and the Hospital Readmission Reduction Program. These programs emphasize cost, quality, and access to create a healthier population.

Prior to April 2015, the US Centers for Medicare & Medicaid Services (CMS) reimbursed physicians based on the Sustainable Growth Rate (SGR) formula.^2^ Medicare used the SGR formula in an attempt to control rising reimbursement costs for physicians caring for Medicare patients.^2^ A payment reduction of more than 20% was scheduled as per this SGR formula.^3^ In April 2015, the SGR formula was repealed with the passage of the Medicare Access and Children’s Health Insurance Program Reauthorization Act (MACRA).^4^

The SGR reimbursement method was repealed because it subjected physicians and health care clinicians to an annual reimbursement cut of up to 21.2%.^3–6^ Following its repeal, two payment systems were put in place to incentivize physicians and clinicians.^5,6^ Those incentive payment programs were: MIPS and Alternative Payment Models.^5,6^

### MIPS: Definition, Four Evaluation Domains, and Eligible and Exempt Practitioners

MIPS is defined as “the default track for clinician participation in the Quality Payment Program (QPP) that adjusts future payment rates for Medicare Part B services based on composite performance across four measures: Quality, Clinical Practice Improvement Activities, Advancing Care Information, and Resource Use.”^6^ These four MIPS measures (or MIPS domains) contribute to a MIPS performance score in Quality, Clinical Practice Improvement Activities, Advancing Care Information, and Resource Use.^6–9^

Table 1 enlists Eligibility and Exemption criteria for clinicians wishing to participate or be exempt from MIPS.^10,11^ Eligible practitioners who are not in advanced Alternative Payment Models are defaulted to the MIPS track.^7^ In 2019, MIPS consolidated three programs: the Physician Quality Reporting System, the Value-Based Payment Modifier, and Meaningful Use of Electronic Health Records into a single program called Clinical Practice Improvement Activities.^7^

**Table 1.** Merit Based Incentive Payment System: Eligible and Exempt Practitioners

**Eligible Practitioners**	**Exempt Practitioners**
MIPS Eligible Practitioners are licensed physicians including doctors of medicine, doctors of osteopathy, osteopathic practitioners, doctors of dental surgery, doctors of dental medicine, doctors of podiatric medicine, doctors of optometry, chiropractors, physician assistants, nurse practitioners, clinical nurse specialists, certified registered nurse anesthetists, clinical psychologists, physical therapists, occupational therapists, qualified speech-language pathologists, qualified audiolo- gists, registered dieticians and nutrition professionals, and clinicians who have billed more than $90,000 in Medicare Part B allowable charges, consult more than 200 Part B patients, and provide 200 or more covered professional services to Medicare Part B patients.	MIPS Exempt Practitioners are those that are not included in any of the eligible clinician types, clinicians who enroll in Medicare for the first time in 2019, who participate in APM, are either a qualifying APM participant or partial qualifying participant, those who are not in a MIPS eligible specialty, clinicians or groups that have billed $90,000 or less in Physician Fee Schedule (PFS) services furnished to Medicare Part B Fee-for-Service (FFS) beneficiaries (including Railroad Retirement Board and Medicare secondary payer), clinicians or groups that have 200 or fewer Medicare Part B FFS beneficiaries, and clinicians or groups that have provided less than 200 covered professional services to Medicare Part B patients.

Abbreviations: MIPs, Merit-based Incentive Payment System; APM, Alternative Payment Model.

Sources: MIPS Eligible Clinician Criteria. https://qpp.cms.gov/mips/how-eligibility-is-determined#mipsEligibleClinicians-2021; MIPS Exemption Criteria for Participation. https://help.practicefusion.com/s/article/What-is-a-MIPS-eligible-clinician-in-2019.

### The National Quality Strategy, Forum, Task Force, and Aims

The National Quality Strategy (NQS) is a nationwide effort to improve the quality of health and health care in the United States.^12^ The National Quality Forum uses the NQS as a roadmap for much of its work.^12^ The National Quality Forum played an important role in shaping the NQS in several ways.^12^

The NQF and several collaborators launched the National Quality Task Force in late 2018.^13^ This task force analyzes the progress of the four stages of the Modern Quality Movement, the fourth stage of which is paying for value.^13^ The NQS pursues three broad aims to guide and assess local, state, and national efforts to improve health and health care quality.^14^ The three NQS aims are: Better Care, Healthy People and Communities, and Affordable Care.^14^

As per NQS, Better Care refers to improving overall health care quality, by making health care more patient-centered, reliable, accessible, and safe.^14^ Healthy People and Communities refers to improving the health of the US population by supporting proven interventions to address behavioral, social, and environmental determinants of health in addition to delivering higher-quality care.^14^ Affordable Care refers to reducing the cost of quality health care for individuals, families, employers, and government.^14^

### Quality Domains

MACRA quality domains are: (1) clinical care, (2) safety, (3) care coordination, (4) patient and caregiver experience, and (5) population health and prevention.^15^ As per the CMS’ Quality Measure Development Plan, the MACRA quality domains, which were mandated for use in MIPS, align with the NQS priority areas and CMS Quality Strategy goals.^15^

CMS strives to clearly align quality measures with these domains to address gaps, drive quality improvement, and ensure the CMS Quality Strategy goals are achieved.^15^ CMS proposed including Affordable Care as a quality domain for MIPS along with the five MACRA quality domains.^15^

The six MIPS quality domains are: (1) Effective Clinical Care (ECC), (2) Efficiency/Cost Reduction (E/CR), (3) Communication and Care Coordination, (4) Person and Caregiver Experience and Outcomes, (5) Patient Safety, and (6) Community and Population Health.^16^

MIPS utilizes these six domains in its Quality Performance Category Percent Score formula to compute points in its scoring system to provide incentives to EPs.^16^ As per the Agency for Healthcare Research and Quality, these six MIPS domains also align with NQS priorities.^17^ The NQS focuses on these six priorities to advance its initiatives in the three broad NQS aims.^17^

The ECC domain aligns with measures incorporating patient preferences, shared decision-making, and outcome measures.^16^ The E/CR domain aligns with decreasing overuse measures by improving efficiencies, cost-effectiveness, and, therefore, affordability of care.^16^ ECC aims to improve the health of the population, whereas E/CR focuses on improving the affordability and cost-effectiveness of treatments.

The MIPS Quality Performance Category factsheet, additionally, mentions seven quality measure types: (1) Structure, (2) Process, (3) Outcome, (4) Intermediate Outcome, (5) Patient Reported Outcome, (6) Efficiency, or (7) Patient Engagement & Patient Experience.^16^

Structure examines the physician’s capacity, systems, and scope to provide high-quality care.^16^ Process looks at the physician’s initiatives to maintain and improve the health of patients.^16^ Outcome measures determine how a physician’s line of treatment affects the patient’s health status.^16^ Intermediate Outcome measures assess specific metrics that contribute to having an outcome.^16^ Patient Reported Outcome measures are the outcomes reported directly by the consumers.^16^ Efficiency measures evaluate the affordability of health care treatments and whether those can be made more cost effective for the patient.^16^ Patient Engagement and Patient Experience measures use direct feedback from patients and their caregivers about their care experience.^16^ This information is usually collected through surveys.^16^

## OBJECTIVES

Given past literature, little is known whether MIPS has had an impact on ECC and E/CR. The broad aim, therefore, is to explore whether MIPS has any influence on the effectiveness of care and affordability of treatments. In the interest of understanding how MIPS affects effectiveness and affordability, this research examines MIPS with regard to ECC and E/ CR domains. In other words, this research aims to understand how MIPS is associated with health care outcomes and affordability, given the two NQS domains, ECC and E/CR.

An extensive review of the literature failed to detect an evaluation of MIPS in the context of QPP measure types and NQS domains. Due to the gap in literature, this topic deserves some investigation to account for and understand how MIPS is associated with effectiveness and affordability of care. This understanding will contribute information regarding the association of MIPS on two specific NQS domains, ECC and E/CR, with quality measure types given the medical and surgical specialties analyzed in this research.

This research has a three-pronged objective. The first objective is to observe the count and percentage of MIPS measures that apply to quality measure types, NQS domains, and specialties. This gives us insight into how NQS domains and quality measure types are distributed in the process of reporting MIPS measures. These results will observe the intricacies, distributions, and percentage allocations of the categories of these variables.

The second objective is to analyze how MIPS measures are reported according to quality measure types and NQS domains by specialties. This information will present annual comparisons based on how reporting MIPS measures varied in the past according to specialties, over the years 2017 through 2020.

The third objective is to identify whether ECC and E/CR are significantly associated with quality measure types and specialties. This information will assist in quantitatively estimating how the two NQS domains associate with quality measure types and specialties in this research.

The purpose of these three objectives is to explore how MIPS’ impact on care effectiveness and affordability is relevant to MIPS as a health care policy.

## METHODS

### Database

The database was retrieved from CMS’ publicly available QPP website for years 2017, 2018, 2019, and 2020.^18–21^ Data pools were obtained from the dropdown menus of quality measure types, specialty measure set, and collection types. These data pools were leveraged to build annual datasets for this research. The 2017 MIPS measure list was the earliest list available.

### Measure Categorization

Quality measure types, NQS domains, and medical/surgical specialties were the categorical variables of this research. Table 2 presents the list of medical and surgical (or clinical) specialties pertaining to this research. These clinical specialties were selected to cover a broad range of specialties and be reasonably inclusive of the different types of specialties. These clinical specialties were leveraged to analyze the effect MIPS would have on the NQS’ ECC and E/CR domains and quality measure types.

**Table 2.** Clinical Specialties Analyzed in This Study

**Medical Specialties**	**Surgical Specialties**
Cardiology Diagnostic Radiology Emergency Medicine Family Medicine Internal Medicine Hospital Medicine or Hospitalists Mental/Behavioral Health Neurology Obstetrics/Gynecology Ophthalmology Otolaryngology Pathology Preventive Medicine Physical Medicine Urology	General Orthopedic Plastic Thoracic Vascular Surgical Specialties

### Dependent and Independent Variables

The ECC and E/CR NQS domains were the primary dependent variables, given their bearing on clinical outcomes and affordability of health care services. QPP measure types and select MIPS clinical specialties were the independent variables.

### Statistical Methods

This research utilized two statistical methods. First, the Pearson’s chi-square test, which is a non-parametric test,^22^ is a cross-tabulated distribution of the variables. It presents distributions^23^ of the categorical variables. This method also shows a contingency table that exhibits statistical significance between the analyzed variables.^22,24^ Second, the Categorical Dependent Variable Method (CDVM)^23^ is used to determine the association between the dependent and independent variables specific to this research. More specifically, the binary logit regression model is used to examine the association between the two dependent variables, ECC and E/CR, in separate regression equations, with the independent variables.

The Pearson’s chi-square test cannot be used to provide any inference about the association between variables.^22^ The CDVM regression method is used to infer an association between the two dependent variables, ECC and E/CR, as the second analytical step to Pearson’s chi-square test. There are several coding methods in regression models, of which the most common method is a dummy variable.^25,26^ The categories of quality measure types and clinical specialties were coded as binary or dummy (0/1) variables.^27^

### Ethics Approval

This research was reviewed and deemed non-human subjects research by the University of Baltimore’s Institutional Review Board (IRB). The software used for statistical analysis was STATA(R) version 14.2 copyrighted 1985-2015 for StataCorp LP.

## RESULTS

Table 3 summarizes quality measure types, NQS domains, and clinical specialties participating and reporting under MIPS. Table 4, columns A through D, summarizes MIPS measure characteristics analyzed by Pearson’s chi-square test for years 2017 through 2020.

**Table 3.** Clinical Specialties, Quality Measure Types and NQS Domains of Select MIPS Measures

	**Measure Name Designated by the CMS QPP**	**Specialty**	**Quality Measure Type**	**NQS Domain**
1	**Radiology: Reminder System for Screening Mammograms.** Percentage of patients undergoing a screening mammogram whose information is entered into a reminder system with a target due date for the next mammogram.	Diagnostic Radiology	Structure	Communication and Care Coordination
2	**Atrial Fibrillation and Atrial Flutter Chronic Anticoagulation Therapy.** Percentage of patients aged 18 years and older with nonvalvular atrial fibrillation or atrial flutter who were prescribed warfarin OR another FDA-approved anticoagulant drug for the prevention of thromboembolism during the measurement period.	Internal Medicine	Process	Effective Clinical Care
3	**Epilepsy: Counseling for Women of Childbearing Potential with Epilepsy.** All female patients of childbearing potential (12 to 44 years old) diagnosed with epilepsy who were counseled or referred for counseling for how epilepsy and its treatment may affect contraception OR pregnancy at least once a year.	Neurology	Process	Effective Clinical Care
4	**Pelvic Organ Prolapse: Preoperative Screening for Uterine Malignancy.** Percentage of patients who are screened for uterine malignancy prior to vaginal closure or obliterative surgery for pelvic organ prolapse.	Obstetrics/ Gynecology	Process	Patient Safety
5	**Primary Open-Angle Glaucoma: Reduction of Intraocular Pressure by 15% OR Documentation of a Plan of Care.** Percentage of patients aged 18 years and older with a diagnosis of primary open-angle glaucoma whose glaucoma treatment has not failed (the most recent intraocular pressure was reduced by at least 15% from the pre-intervention level) OR if the most recent intraocular pressure was not reduced by at least 15% from the pre-intervention level, a plan of care was documented within 12 months.	Ophthalmology	Outcome	Communication and Care Coordination
6	**Urinary Incontinence: Plan of Care for Urinary Incontinence in Women Aged 65 Years and Older.** Percentage of female patients aged 65 years and older with a diagnosis of urinary incontinence with a documented plan of care for urinary incontinence at least once within 12 months.	Internal Medicine	Process	Person- and Caregiver-Centered Experience and Outcomes
7	**Acute Otitis Externa: Systemic Antimicrobial Therapy—Avoidance of Inappropriate Use.** Percentage of patients aged 2 years and older with a diagnosis of acute otitis externa who were not prescribed systemic antimicrobial therapy.	Internal Medicine	Structure	Efficiency and Cost Reduction
8	**Urinary Incontinence: Plan of Care for Urinary Incontinence in Women Aged 65 Years and Older.** Percentage of female patients aged 65 years and older with a diagnosis of urinary incontinence with a documented plan of care for urinary incontinence at least once within 12 months.	Obstetrics/ Gynecology	Process	Person- and Caregiver-Centered Experience and Outcomes
9	**Overuse of Neuroimaging for Patients with Primary Headache and a Normal Neurological Examination.** Percentage of patients with a diagnosis of primary headache disorder for whom advanced brain imaging was not ordered.	Neurology	Efficiency	Efficiency and Cost Reduction
10	**Preventive Care and Screening: Tobacco Use: Screening and Cessation Intervention.** Percentage of patients aged 18 years and older who were screened for tobacco use one or more times within 24 months AND who received tobacco cessation intervention if identified as a tobacco user.	Plastic Surgery	Process	Community/ Population Health
11	**Perioperative Care: Selection of Prophylactic Antibiotic—First OR Second-Generation Cephalosporin.** Percentage of surgical patients aged 18 years and older undergoing procedures with the indications for a first OR second-generation cephalosporin prophylactic antibiotic who had an order for a first OR second- generation cephalosporin for antimicrobial prophylaxis.	General Surgery	Process	Patient Safety
12	**Advance Care Plan.** Percentage of patients aged 65 years and older who have an advance care plan or surrogate decision-maker documented in the medical record or documentation in the medical record that an advance care plan was discussed but the patient did not wish or was not able to name a surrogate decision- maker or provide an advance care plan.	Vascular Surgery	Process	Communication and Care Coordination
13	**Documentation of Current Medications in the Medical Record.** Percentage of visits for patients aged 18 years and older for which the MIPS eligible clinician attests to documenting a list of current medications using all immediate resources available on the date of the encounter. This list must include ALL known prescriptions, over-the-counters, herbals, and vitamin/mineral/dietary (nutritional) supplements AND must contain the medications’ name, dosage, frequency and route of administration.	Thoracic Surgery	Process	Patient Safety
14	**Quality of Life Assessment for Patients with Primary Headache Disorders**. Percentage of patients with a diagnosis of primary headache disorder whose health-related quality of life was assessed with a tool(s) during at least two visits during the 12 month measurement period AND whose health related quality of life score stayed the same or improved.	Neurology	Patient-Reported Outcome	Effective Clinical Care
15	**Diabetes: Hemoglobin A1c Poor Control (>9%).** Percentage of patients 18-75 years of age with diabetes who had hemoglobin A1c >9.0% during the measurement period.	Internal Medicine	Intermediate Outcome	Effective Clinical Care

Abbreviations: CMS, Centers for Medicare & Medicaid Services; MIPS, Merit-based Incentive Payment System; NQS, National Quality System; Quality Payment Program, QPP.

**Table 4.** MIPS Measure Characteristics Analyzed by Pearson’s Chi-Square Test (Years 2017 through 2020)

	**A**		**B**			**C**			**D**		
**Measure Characteristics**	**2017**		**2018**		***P*-value**	**2019**		***P*-value**	**2020**		***P*-value**
	**(n=193)**		**(n=210)**			**(n=208)**			**(n=194)**		
	**Count (%)**										
**A. Quality Measure Types**											
Structure	2	1.0	1	0.5	0.000	1	0.5	0.000	1	0.5	0.000
Process	173	89.6	197	93.8		195	93.8		182	93.8	
Outcome	5	2.6	2	1.0		1	0.5		1	0.5	
Intermediate Outcomes	10	5.2	7	3.3		8	3.8		8	4.1	
Patient-Reported Outcomes	0	0.0	0	0.0		1	0.5		1	0.5	
Efficiency	3	1.6	3	1.4		2	1.0		1	0.5	
**B. NQS Domains**											
Effective Clinical Care	53	27.5	55	26.2	0.258	48	23.1	0.000	42	21.6	0.028
Efficiency and Cost Reduction	7	3.6	8	3.8		8	3.8		7	3.6	
Patient Safety	36	18.7	45	21.4		46	22.1		46	23.7	
Communication and Care Coordination	30	15.5	38	18.1		38	18.3		35	18.0	
Person- and Caregiver-centered Experience and Outcomes	9	4.7	8	3.8		8	3.8		4	2.1	
Community and Population Health	58	30.1	56	26.7		60	28.8		60	30.9	
**C. Select MIPS Specialties**											
Cardiology	8	4.1	8	3.8	0.000	7	3.4	0.000	7	3.6	0.000
Diagnostic Radiology	8	4.1	8	3.8		8	3.8		8	4.1	
Emergency Medicine	10	5.2	7	3.3		7	3.4		4	2.1	
Family Medicine	21	10.9	25	11.9		25	12.0		24	12.4	
Hospitalists	8	4.1	4	1.9		4	1.9		3	1.5	
Internal Medicine	20	10.4	20	9.5		22	10.6		21	10.8	
Mental Health	7	3.6	6	2.9		6	2.9		6	3.1	
Neurology	9	4.7	9	4.3		11	5.3		11	5.7	
Obstetrics/Gynecology	12	6.2	13	6.2		14	6.7		14	7.2	
Ophthalmology	10	5.2	8	3.8		7	3.4		6	3.1	
Otolaryngology	9	4.7	13	6.2		13	6.3		12	6.2	
Pathology	8	4.1	8	3.8		5	2.4		5	2.6	
Physical Medicine	8	4.1	10	4.8		10	4.8		8	4.1	
Preventive Medicine	14	7.3	17	8.1		17	8.2		17	8.8	
Urology	6	3.1	10	4.8		10	4.8		9	4.6	
General Surgery	7	3.6	8	3.8		8	3.8		7	3.5	
Orthopedic Surgery	8	4.1	15	7.1		15	7.2		13	6.6	
Plastic Surgery	7	3.6	5	2.4		5	2.4		5	2.5	
Thoracic Surgery	7	3.6	7	3.3		6	2.9		6	3.0	
Vascular Surgery	6	3.1	9	4.3		8	3.8		8	4.0	

Abbreviations: MIPS, Merit-based Incentive Payment System; NQS, National Quality System.

Of the 2017 MIPS measures selected for the research, when classified by quality measure types, 2 (1.04%) assessed structure, 173 (89.64%) assessed process, 5 (2.59%) assessed outcome, 10 (5.18%) assessed intermediate outcome, and 3 (1.55%) assessed efficiency (Table 4). For the 2017 NQS domains, 53 (27.46%) focused on ECC, 7 (3.63%) focused on E/CR, 36 (18.65%) focused on patient safety, 30 (15.54%) focused on communication and care coordination, 9 (4.66%) focused on person- and caregiver-centered experience and outcomes, and 58 (30.05%) focused on community and population health. Table 4 also summarizes the count and percentages of the analyzed specialties.

When compared with 2017 measures, the quality measure type changed significantly with more process measures (93.81% vs 89.64% [*P*=0.000]) in 2018 versus 2017. Similarly, when compared with 2017, 2018 specialty measures were statistically significant. There was no significant change in the NQS domain categories between 2017 and 2018 (Table 4).

When compared with 2018 measures, the quality measure types changed significantly with fewer process measures (93.75% vs 93.81% [*P*=0.000]) in 2019 versus 2018. Similarly, when compared with 2018, the 2019 specialty measures had statistically significant differences. There was a statistically significant distribution between the NQS domains with fewer ECC measures (23.08% vs 26.19% [*P*=0.000]) in 2019 versus 2018 (Table 4).

Compared with 2019, the 2020 clinical specialty measures were statistically significant. When compared with 2019 measures, the quality measure type changed significantly with less process measures (93.81% vs 93.75% [*P*=0.000]) in 2020 versus 2019. There was no statistical significance in the NQS domain categories in 2020 compared with 2019 (Table 4).

**Tables**5A and 5B summarize MIPS measure characteristics by clinical specialties for year 2019. When quality measure types were assessed, statistical significance was observed for the analyzed specialties. Orthopedic surgery had the highest number of process measures, 15 (100%), and obstetrics/gynecology had the lowest number of intermediate outcome measures, 1 (7.14%). No statistical significance was observed between the NQS domains and analyzed specialties.

**Table 5A.** MIPS Characteristics by Specialty Type, 2019, Measure Types

**Measure Characteristics**							
**Specialties**	**Structure**	**Process**	**Outcome**	**Intermediate Outcomes**	**Patient-Reported Outcomes**	**Efficiency**	***P*-value**
Cardiology (n=7)	0	6 (85.7%)	0	1 (14.3%)	0	0	
Diagnostic Radiology (n=8)	1 (12.5%)	7 (87.5%)	0	0	0	0	
Emergency Medicine (n=7)	0	5 (71.4%)	0	0	0	2 (28.5%)	
Family Medicine (n=25)	0	23 (92%)	0	2 (8%)	0	0	
Hospitalists (n=4)	0	4 (100%)	0	0	0	0	
Internal Medicine (n=22)	0	20 (90.9%)	0	2 (9.1%)	0	0	
Mental Health (n=6)	0	6 (100%)	0	0	0	0	
Neurology (n=11)	0	10 (90.9%)	0	0	1 (9.1%)	0	
Obstetrics/Gynecology (n=14)	0	13 (92.9%)	0	1 (7.1%)	0	0	
Ophthalmology (n=7)	0	6 (85.7%)	1 (14.3%)	0	0	0	0.001
Otolaryngology (n=13)	0	13 (100%)	0	0	0	0	
Pathology (n=5)	0	5 (100%)	0	0	0	0	
Physical Medicine (n=10)	0	10 (100%)	0	0	0	0	
Preventive Medicine (n=17)	0	16 (94.1%)	0	1 (5.8%)	0	0	
Urology (n=10)	0	10 (100%)	0	0	0	0	
General Surgery (n=8)	0	8 (100%)	0	0	0	0	
Orthopedic Surgery (n=15)	0	15 (100%)	0	0	0	0	
Plastic Surgery (n=5)	0	5 (100%)	0	0	0	0	
Thoracic Surgery (n=6)	0	6 (100%)	0	0	0	0	
Vascular Surgery (n=8)	0	7 (87.5%)	0	1 (12.5%)	0	0	

Abbreviations: MIPS, Merit-based Incentive Payment System; NQS, National Quality System.

**Table 5B.** MIPS Measure Characteristics by Specialty Type, 2019, NQS Domains Measure Characteristics

Specialties	Effective Clinical Care	Patient Safety	Efficiency and Cost Reduction	Communication and Care Coordination	Community and Population Health	Person- and Caregiver-centered Experience and Outcomes	*P*-value
Cardiology (n=7)	2 (28.8%)	1 (14.3%)	0	1 (14.3%)	3 (42.8%)	0	
Diagnostic Radiology (n=8)	4 (50%)	1 (12.5%)	1 (12.5%)	2 (25%)	0	0	
Emergency Medicine (n=7)	3 (42.2%)	0	3 (42.8%)	0	1 (14.2%)	0	
Family Medicine (n=25)	10 (40%)	3 (12%)	1 (4%)	3 (12%)	6 (25%)	2 (8%)	
Hospitalists (n=4)	0	2 (50%)	1 (25%)	1 (25%)	0	0	
Internal Medicine (n=22)	8 (36.4%)	3 (13.6%)	1 (4.5%)	3 (13.6%)	6 (27.3%)	1 (4.5%)	
Mental Health (n=6)	0	2 (33.3%)	0	0	4 (66.7%)	0	
Neurology (n=11)	2 (18.1%)	3 (27.3%)	2 (18.2%)	2 (18.2%)	3 (27.3%)	0	
Obstetrics/Gynecology (n=14)	4 (28.6%)	3 (21.4%)	0	1 (7.1%)	5 (35.7%)	1 (7.1%)	
Ophthalmology (n=7)	3 (42.8%)	1 (14.3%)	0	2 (28.6%)	1 (14.3%)	0	
Otolaryngology (n=13)	1 (7.7%)	4 (30.8%)	1 (7.7%)	2 (15.4%)	5 (38.5%)	0	0.158
Pathology (n=5)	2 (40%)	0	0	3 (60%)	0	0	
Physical Medicine (n=10)	0	2 (20%)	0	4 (40%)	3 (30%)	1 (10%)	
Preventive Medicine (n=17)	5 (29.4%)	2 (11.8%)	0	3 (17.6%)	6 (35.3%)	1 (5.9%)	
Urology (n=10)	1 (10%)	3 (30%)	0	2 (20%)	3 (30%)	1 (10%)	
General Surgery (n=8)	0	3 (37.5%)	0	2 (25%)	3 (37.5%)	0	
Orthopedic Surgery (n=15)	1 (6.7%)	4 (26.7%)	0	5 (33.3%)	4 (26.7%)	1 (6.7%)	
Plastic Surgery (n=5)	0	3 (60%)	0	0	2 (40%)	0	
Thoracic Surgery (n=6)	0	3 (50%)	0	1 (16.7%)	2 (33.3%)	0	
Vascular Surgery (n=8)	1 (12.5%)	3 (37.5%)	0	1 (12.5%)	3 (37.5%)	0	

Table 6 summarizes the results of the CDVM for the two NQS domains, ECC and E/CR, when regressed with quality measure types and clinical specialties, for years 2017 through 2020.

**Table 6.** Results of the Categorical Dependent Variable Method Binary Logit Regression Analyzed for NQS Domains When Regressed with Quality Measure Types and Clinical Specialties for Years 2017 through 2020

**NQS Domain Type When Tested with QPP Measure Types and Select MIPS Specialties**	**2017**			**2018**			**2019**			**2020**		
	**(n=193)**			**(n=210)**			**(n=208)**			**(n=194)**		
	**Coefficient *P*-value (95% CI)**											
**Independent Variables**	**A. Effective Clinical Care**											
**Quality Measure Types**												
Structure	-0.94	0.00	(-1.32 -0.56)	-1.04	0.00	(-1.56 -0.51)	-0.99	0.00	(-1.73 -0.26)	-0.93	0.00	(-1.44 -0.43)
Process	-0.86	0.00	(-1.03 -0.68)	-0.87	0.00	(-1.06 -0.67)	-0.83	0.00	(-1.35 -0.30)	-0.94	0.00	(-1.26 -0.62)
Outcome	-0.82	0.00	(-1.09 -0.55)	-0.54	0.00	(-0.93 -0.14)	-0.99	0.00	(-1.73 -0.26)	-0.95	0.00	(-1.44 -0.45)
Efficiency	-1.02	0.00	(-1.34 -0.70)	-1.04	0.00	(-1.38 -0.69)	-0.99	0.00	(-1.63 -0.36)	-0.99	0.00	(-1.44 -0.55)
Intermediate Outcome	omitted (referent category)			omitted (referent category)			0.01	0.95	(-0.53 0.56)	0.04	0.93	(-0.32 0.33)
Patient-Centered Outcome	N/A			N/A			omitted (referent category)			omitted (referent category)		
**Select MIPS Specialties**												
Medical	omitted (referent category)			omitted (referent category)			omitted (referent category)			omitted (referent category)		
Surgical	-0.12	0.22	(-0.32 0.07)	-0.14	0.02	(-0.26 -0.01)	-0.14	0.01	(-0.31 0.03)	-0.04	0.04	(-0.15 0.06)
**Intercept**	1.86			1.88			1.83			1.94		
**Probability of Cluster**	0.00			0.00			0.00			0.00		
**R^2^**	0.77			0.76			0.75			0.91		
**Pseudo R^2^**	0.75			0.75			0.73			0.90		
**Independent Variables**	**B. Efficiency and Cost Reduction**											
**Quality Measure Types**												
Structure	0.04	0.78	(-0.27 0.36)	-0.02	0.89	(-0.46 0.41)	-0.03	0.90	(-0.68 0.61)	-0.05	0.90	(-0.72 0.62)
Process	0.11	0.13	(-0.03 0.25)	0.09	0.27	(-0.07 0.25)	0.14	0.56	(-0.34 0.63)	0.17	0.51	(-0.34 0.68)
Outcome	-0.02	0.86	(-0.24 0.20)	-0.02	0.86	(-0.35 0.30)	-0.03	0.91	(-0.68 0.61)	-0.05	0.91	(-0.72 0.62)
Efficiency	0.98	0.00	(0.71 1.25)	0.97	0.00	(0.68 1.25)	0.99	0.00	(0.40 1.59)	0.99	0.00	(0.27 1.72)
Intermediate Outcome	omitted (referent category)			omitted (referent category)			0.01	0.95	(-0.50 0.53)	0.01	0.95	(-0.52 0.55)
Patient-Centered Outcome	N/A			N/A			omitted (referent category)			omitted (referent category)		
**Select MIPS Specialties**												
Medical	omitted (referent category)			omitted (referent category)			omitted (referent category)			omitted (referent category)		
Surgical	-0.09	0.24	(-0.26 0.06)	-0.09	0.05	(-0.20 0.01)	-0.12	0.14	(-0.28 0.04)	-0.13	0.15	(-0.31 0.05)
**Intercept**	-1.08			-0.08			-0.14			-0.16		
**Probability of Cluster**	0.00			0.00			0.00			0.00		
**R^2^**	0.46			0.40			0.29			0.22		
**Pseudo R^2^**	0.42			0.37			0.24			0.15		

Abbreviations: MIPS, Merit-based Incentive Payment System; NQS, National Quality System; Quality Payment Program, QPP.

The binary logit regression model for year 2017 estimated that for every one unit increase in the outcome measure (as a nested subcategory of quality measure type as an independent variable), the odds that ECC is significantly 0.82 times as small as the odds that this measure is not included in this domain, when all the other variables are held constant.

Similarly, the regression models for years 2018, 2019, and 2020 estimated that for every one unit increase in the outcome measure, the odds that ECC is significantly 0.54, 0.99, and 0.95 times as small as the odds that these observations are not included in this domain, respectively, when all the other variables are held constant.

The binary logit regression model for 2017 through 2020 estimated that for every one unit increase in the outcome measure, the odds that E/CR, although not statistically significant (from the *P*-values), is the indicated unit times as small as the odds that these observations are not included in this domain, when all the other variables are held constant.

## DISCUSSION

The analysis exhibited both growth and scope for advancement of MIPS in terms of measure selection. MIPS measures have expanded in reporting more quality improvement measures by including different measure types across clinical specialties.

The number of MIPS process measures increased by 19 between 2017 and 2018. Those additional 2018 process measures for MIPS specialties are listed in Table 7.

**Table 7.** Examples of Additional 2018 MIPS Measures Compared to 2017

**#**	**MIPS Measure Names**	**Specialty**	**Measure Type**	**NQS Domain**
1	Acute Otitis Externa: Topical Therapy	Family Medicine	Process	ECC
2	Colorectal Cancer Screening	Preventive Medicine	Process	ECC
3	Communication with the Physician or Other Clinician Managing Ongoing Care Post-Fracture for Men and Women Aged 50 Years and Older	Family Medicine Orthopedic Surgery	Process	CCC
4	Falls: Plan of Care	Otolaryngology, Physical Medicine, Preventive Medicine and Orthopedic Surgery	Process	CCC
5	Falls: Risk Assessment	Otolaryngology, Physical Medicine, Preventive Medicine and Orthopedic Surgery	Process	PS
6	Medication Reconciliation Post-discharge	General Surgery, Orthopedic Surgery	Process	CCC
7	Osteoporosis Management in Women Who Had a Fracture	Orthopedic Surgery	Process	ECC
8	Pain Assessment and Follow-up	Urology, Orthopedic Surgery	Process	CCC
9	Pelvic Organ Prolapse: Preoperative Screening for Uterine Malignancy	Obstetrics/Gynecology Urology	Process	PS
10	Perioperative Anti-platelet Therapy for Patients Undergoing Carotid Endarterectomy	Vascular Surgery	Process	ECC
11	Perioperative Care: Selection of Prophylactic Antibiotic—First OR Second Generation Cephalosporin	Vascular Surgery	Process	PS
12	Perioperative Care: Venous Thromboembolism Prophylaxis when Indicated in all Patients	Urology, Vascular Surgery	Process	PS
13	Pneumococcal Vaccination Status for Older Adults	Family Medicine, Orthopedic Surgery	Process	CPH
14	Preventive Care and Screening: Body Mass Index Screening and Follow-up Plan	Urology	Process	CPH
15	Preventive Care and Screening: Influenza Immunization	Otolaryngology	Process	CPH
16	Preventive Care and Screening: Screening for Depression and Follow-up Plan	Orthopedic Surgery	Process	CPH
17	Preventive Care and Screening: Screening for High Blood Pressure and Follow-up Documented	Preventive Medicine	Process	CPH
18	Screening for Osteoporosis for Women Aged 65-85 Years of Age	Family Medicine	Process	ECC
19	Urinary Incontinence: Assessment of Presence or Absence of Urinary Incontinence in Women Aged 65 Years and Older	Family Medicine	Process	ECC

Abbreviations: CCC, Communication and Care Coordination; CPH, Community and Population Health; ECC, Effective Clinical Care; MIPS, Merit-based Incentive Payment System; PS, Patient Safety.

In connection to the scope for advancement, MIPS had a limited number of measure types that focused on outcomes. The number of priority outcome measures (PROMs) reported for MIPS Medicare Part B measures in 2017, 2018, 2019, and 2020 were 7, 3, 2 and 2, respectively.^18–21^

Outcome measures may need to be included more because they “often directly measure events with the clearest importance in clinical management and treatment.”^27^ Most physicians and patients are interested in high-quality outcomes following treatment and surgical procedures. These outcomes include, for example, survival rates following major surgeries, decreasing hospital readmission rates, reducing preventable hospitalizations, preserving the quality of life after medical treatments, and controlling pneumonia and heart failure, to name a few.^28^

Notably, the Agency for Healthcare Research and Quality has emphasized that “outcome measures reflect the impact of healthcare services or interventions on the health statuses of patients.”^29^ Additionally, MIPS has a “priority focus on outcome measures, [which are] measures that provide new measure options within a topped-out specialty area or measures that are relevant for specialty providers.”^30^ Kessell et al have importantly noted that “health outcomes are essential for assessing quality of care and include a variety of health statuses.”^31^

Increasing the number of outcome measures may broaden the scope for advancing health statuses. Addressing not only outcomes but all six of the enlisted NQS domains is a substantial initiative. These NQS domains are only one of the many ways to address the Institute of Medicine’s six aims for an optimized health care system. The Institute of Medicine’s six aims—safe, efficient, effective, patient-centered, timely and equitable—are listed in the 2001 *Crossing the Quality Chasm* report.^27,32^

In MIPS, outcome measures are the “gold standard” in measuring quality.^33^ Outcome measures show how a health care service or intervention influences the health status of patients.^33^ All seven quality measure types (efficiency, intermediate outcome, outcome, patient-reported outcome, patient engagement and experience, process, and structure) include high-priority measures.^33^ High-priority measures are not an additional measure type by itself but an incorporated aspect within the seven quality measure types.^33^

MIPS is the most expansive pay for performance program to date.^34^ In a national survey analyzing MIPS’ impact on value of care, 19% to 24% of survey respondents believed MIPS would, in fact, reduce the value of care.^34^ In the same vein, physicians were wary of how MIPS would affect the value of care.^34^

Under MIPS, provider reimbursement is determined by two primary factors: a composite score and program participation that provides fiscal incentives ranging from 4% to 9%—depending on the year of reporting.^8^ The reporting of MIPS potentially creates administrative burdens given its “extensive and complex quality reporting requirements” that are intensive and time-consuming.^35^

These additional reporting requirements may come at the cost of diverting time and resources from that allotted to direct patient care, thereby negatively impacting patient and provider satisfaction scores.^36^ A negative impact on patient and/or provider satisfaction scores may extend a similar impact on the quality of care.

Lower patient satisfaction may be the likely outcome of lower standards of care just as excessive diagnostic testing may potentially result in less satisfied patients.^36^ Green et al (2017) found that provider incentive payment programs increased the number of incentivized measures included in the MIPS cohort in their simulated experiment.^36^ They observed that unnecessary diagnostic testing puts the patient at a greater risk for overtreatment, likely increasing the cost of health care.^36^ In their experiment, clinicians incentivized under MIPS were more likely to overtreat and be paid more resulting in additional system costs, thereby stressing an already overstretched health care system.^36^

### Limitations and Strengths

This research is specific only in its applications to MIPS as a policy. It may not extend its applications to other pay-for-performance incentive-based policies. Findings are limited only to the clinical specialties that were analyzed for this research.

This research describes two NQS domains in relation to clinical outcomes and affordability of care. It presents the effect of MIPS as an aggregate overall policy and not at the granular domain levels that contribute to the MIPS final score.

The Pearson’s chi-square test is specific to the sample size.^37^ This chi-square test is not recommended if the sample size is less than 50 units.^37^ The limitation in methodology is not applicable to this research because the sample size is a minimum of 193 for each of the analyzed years.

The observations of this research, essentially, the quality measure types, NQS domains, and clinical specialties, were distinct units and had no overlaps. The Pearson’s chi-square test assumes random sampling and that the observations must be randomly selected from the total population.^37^ The observations were, in fact, of no specific or sequential order of selection when analyzed for this research.

The CDVM is not relevant to showing any changes in levels and trends in research observations. The purpose of this research was not to observe changes in levels and trends but to understand the distributions and association of the analyzed variables. This was to understand this policy with regard to effectiveness and affordability of care. The CDVM model is limited in its analysis to categorical variables, which this research has employed.

Considering the strengths of this research and its statistical methods, this research analyzed each MIPS reporting year starting in 2017, the first year for reporting MIPS measures. This information may be useful in providing historical base data for comparing future values while MIPS is in practice in forthcoming years.

The association between the categorical variables of this research was analyzed by leveraging two statistical methods, Pearson’s chi-square test and binary logit regression model. The Pearson’s chi-square test does not give information about the strength or associations between the analyzed variables, which is why a regression model was leveraged. The regression model made it feasible to quantitatively examine the associations of the categories of the analyzed variables.

### Future Work

It will be interesting to observe the effects of MIPS leveraging a longitudinal panel regression model. Panels could be constructed to include quality measure types, the years of analysis, and clinical specialties for regressing with the dependent variable. Research leveraging NQS domains as the dependent variable analyzed with independent variables would be another avenue to further research based on this policy utilizing a longitudinal panel regression model.

### Practice and Policy Implications

MIPS may be discontinued due to its heavy administrative burdens and diversion of time and resources from direct patient care.^38^ MIPS has had little positive impact on both physician and patient satisfaction.^38^ Consequently, MIPS may have failed to improve the patient experience of care.42 Moreover, MIPS may not measure the broader aspects of health care quality and even risk worsening existing health disparities.^38^

MIPS has specific reporting requirements allocated according to a defined formula.^8^ This formula determines the pay for performance and bonus point incentive payments for specialties and hospitals reporting under MIPS. In 2021, this formula showed that MIPS has four domains (percentage allocations) of quality (40%), cost (20%), improvement activities (15%), and promoting interoperability (25%).^39^

From a policy implications perspective, MIPS has the potential to increase the percentage allocation to report more quality outcome measures in its incentive-score formula. Adjusting this incentive formula to reflect increased reporting of more quality outcome measures would also support reporting more metrics that align with PROMs. This formula adjustment would create and record more quality outcomes in treatment.

Increased quality measure reporting may permit and facilitate clinical specialties and participating hospitals to redesign, innovate, implement, report, and be recognized for more MIPS quality outcome measures. And, in that case, MIPS could be instrumental in reactivating new quality improvement initiatives.

## CONCLUSION

This research analyzed MIPS through the lenses of health care outcomes and affordability pertaining to ECC and E/CR. MIPS has a priority focus on outcome measures. In addition to being reflective of health statuses of patients, outcome measures are the “gold standard” in measuring high-quality health care.^33^

These outcome measures signify the patients’ health statuses—the result of the treatment, procedure, or intervention. Most patients and physicians are interested in outcome measures of health statuses following treatment, surgery, and intervention.

Policy makers and analysts may need to review the components of MIPS and its percentage requirements of reporting. It may be helpful to preserve MIPS’ existing areas and expand areas where reporting more outcome measures could be showcased.
